# Adaptive tracking control for nonlinear systems with uncertain control gains and its application to a TCP/AQM network

**DOI:** 10.1038/s41598-023-41799-7

**Published:** 2023-09-05

**Authors:** Ziheng Zhang, Zhongtao Liu, Na Liu, Weikai He, Fuchun Yang

**Affiliations:** 1 School of Information Science and Electrical Engineering, Shandong Jiaotong University, Haitang Road 5001, Jinan, 250357 China; 2 Shandong Shansen CNC Technology Co., Ltd, Yuanda Road, Tengzhou, 277500 Shandong China; 3 Shandong Jianzhu University Architecture and Urban Planning Design Institute, Jinan, China; 4 School of Aeronautics, Shandong Jiaotong University, Haitang Road 5001, Changqing District, Jinan, 250000 China; 5 School of Mechanical Engineering, Shandong University, Jingshi Road 17923, Jinan, 250061 China

**Keywords:** Electrical and electronic engineering, Information technology

## Abstract

This paper is concerned with the adaptive tracking control problem for nonlinear systems with virtual control coefficients including known and unknown items. The known items are employed for controller design directly, such that more information is utilized to achieve better performance. To deal with the unknown items, a novel real control law is firstly constructed by introducing an auxiliary system. The proposed controller is designed and applied to an uncertain TCP/AQM network system, which guarantees the practical boundedness of all the signals in the closed-loop system. Finally, the effectiveness and practicability of the developed control strategy are validated by simulation results.

## Introduction

Recent years, the adaptive tracking control for nonlinear systems has been a popular technique in science and engineering. There have been a lot of research results in this area^1–6^, especially since the backstepping method was proposed^7^. In view of finite-time control and constrained input/output, two adaptive tracking controllers were designed for marine surface vehicles under external disturbances^4,6^ . An adaptive control strategy was presented by considering three event-triggered control schemes^5^. Combining state constraints and input deadzone, an adaptive finite-time tracking control approach was proposed^1^. By introducing an improved performance function, an adaptive tracking control method was studied based on funnel control^2^. For high-order fully actuated nonlinear systems with unknown parameters, an adaptive controller was introduced using tuning functions to remove overparametrization^3^. Considering systems with uncertain nonlinear functions, an fuzzy control strategy was given^8^. The usual control problems, such as external perturbations, uncertain parameters and nonlinear functions are handled in the above papers. It needs to highlight that the virtual control coefficients are assumed to be 1^5,8^, known constants^1,4,6^ or known nonlinear functions^2,3^.

However, virtual control coefficients may not be completely known in practical applications. There have been several approaches that can be used to construct controllers for nonlinear systems with uncertain virtual control coefficients. Nussbaum gain, one of the most popular techniques, was firstly proposed^9^, which employed even Nussbaum functions to design tracking controllers for nonlinear systems with unknown constant virtual control coefficients^9–13^. Ge et al. addressed the control problem for nonlinear systems with unknown time-varying virtual control coefficients^14^. Furthermore, the Nussbaum gain technique was generalized to nonlinear systems, whose virtual control coefficients were unknown nonlinear functions of system states^15^. There also have been many relevant achievements^16–20^. Moreover, virtual control coefficients could be handled according to the inequality $\left| x\right| -x/\sqrt{x^{2}+\lambda ^{2}}<\lambda$, where *x* is any real number and $\lambda >0$^21,22^. In addition, by approximating uncertainties of nonlinear systems via fuzzy logic^23,24^ or neural network^25,26^, the control laws $\alpha _{i}$s were constructed to ensure $z_{i}\alpha _{i}\le 0$ such that virtual control coefficients could be replaced by their lower bounds, where $z_{i}=x_{i}-\alpha _{i-1}$ with $x_{i}$ being system state, $i=1$, 2, $\ldots$, *n*. Nevertheless, the virtual control coefficients of nonlinear systems studied by above strategies are always assumed to take values in nonzero closed intervals. And the bounds of virtual control coefficients are chosen to small in simulations, which are difficult to implement when the bounds of virtual control coefficients are big.

The research on active queue management (TCP/AQM) systems has been a hot topic^27–32^. Recently, the adaptive control technique was applied to congestion control for TCP/AQM networks. An adaptive congestion controller was developed for a TCP/AQM network based on a new finite-time performance function^33^. Considering fuzzy logic and funnel control, an adaptive congestion control approach was given for a TCP/AQM network^34^. In view of barrier Lyapunov and neural network, the adaptive TCP/AQM network congestion control was studied^35^. Furthermore, aiming at a novel system model with the uncertain link bandwidth, which was supposed to be known^33–35^, two adaptive TCP network congestion controllers were designed^36,37^. However, it is noted that the link bandwidth *C* is unknown, but the round-trip time *R*(*t*) is taken to be known, which is a function of *C* in^36,37^. Obviously, this is contradictory.

Encouraged by the previous results, this paper is devoted to the adaptive control problem for nonlinear systems with unknown virtual control coefficients and its application to TCP/AQM network systems. Firstly, both controller design and stability analysis are achieved for a class of uncertain nonlinear systems. Furthermore, the proposed control method is applied to a TCP/AQM network, which guarantees the practical boundness of all the signals in the closed-loop system. Finally, the effectiveness of the developed controller is validated by two simulation examples.

The main contributions of this paper are stated as

1. The virtual control coefficients of nonlinear systems are formulated by known and unknown terms so that the bounds of virtual control coefficients are smaller than those expressed as one unknown item in^9–26^. In applications, virtual control coefficients of many real systems are partially known. Therefore, It is feasible to separate virtual control coefficients into known constants and unknown functions by selecting suitable nominal values of system states according to practical requirements. This is demonstrated by the application to a TCP/AQM network.
2. By defining the variable ${\dot{\Psi }}_{r}$ in (23), a new auxiliary system (21) is designed to compensate the items due to the unknown parts of virtual control coefficients, and a novel control law (17) is developed, which ensures the practical bounded control of nonlinear systems.
3. Compared to these adaptive TCP/AQM network congestion control schemes^33–37^, not only the link bandwidth *C* but also the round-trip time *R*(*t*) are considered to be unknown. This is an improvement on the methods in^33–35^. Simultaneously, the contradiction existing in^36,37^ is excluded.

The outline of the paper is organized as: second section presents the problem formulation and preliminaries. The control design and stability analysis is given in next section, and its application to a TCP/AQM network follows in next section. Finally, the simulation results and conclusion are shown in section V and VI, respectively.

## Problem formulation and preliminaries

The following dynamics of an uncertain TCP/AQM network is considered^33–37^.

1 $$\begin{aligned} \left\{ \begin{array}{l} {\dot{x}}_{1}=\frac{{CN}}{x_{1}+CT_{p}}x_{2}-C \\ {\dot{x}}_{2}={-}\frac{Cx_{2}^{2}}{2\left( x_{1}+CT_{p}\right) }u_{r}+\frac{C}{ x_{1}+CT_{p}} \\ y^{*}=x_{1} \end{array} \right. \end{aligned}$$

where $x_{1}$ and $x_{2}$ are the queue length of router and TCP window size, respectively, $T_{p}$ is the propagation delay, *N* is the TCP network load, both known, *C* is the link bandwidth, which is an unknown constant, $u_{r}$ is the packet drop probability.

### *Remark 1*

From the actual physical connotations, it is reasonable to assume that $T_{p}>0$, $N>0$, $x_{1}\in \left[ x_{1m},x_{1M}\right]$, $x_{2}\in \left[ 1,x_{2M}\right]$,$\ {C\in }\left[ C_{m},C_{M}\right]$ and $u_{r}\in \left[ 0,1\right]$ with $x_{1m}$, $x_{1M}$, $x_{2M}$, $C_{m}$ and $C_{M}$ being positive constants. Thus, it is known that there is no singularity in the virtual control coefficients.

Firstly, $\frac{C}{x_{1}+CT_{p}}$ is transformed into the following form by introducing $x_{10}$, $C_{0}$, which are the nominal values of $x_{1}$ and *C*.

2 $$\begin{aligned} \frac{C}{x_{1}+CT_{p}}=\, &  \frac{C_{0}}{x_{10}+C_{0}T_{p}}+\frac{C}{ x_{1}+CT_{p}}-\frac{C_{0}}{x_{10}+C_{0}T_{p}} \nonumber \\= & \,p_{0}+\Delta \end{aligned}$$

where

3 $$\begin{aligned} p_{0}=\frac{C_{0}}{x_{10}+C_{0}T_{p}} \end{aligned}$$

and

4 $$\begin{aligned} \Delta =\frac{C}{x_{1}+CT_{p}}-p_{0} \end{aligned}$$

### Assumption 1

Suppose that the queue length of router $x_{1}$ and link bandwidth *C* are bounded.

According to Assumption 1, it is shown that $\Delta$ is bounded, which is expressed as

5 $$\begin{aligned} \Delta \le \Delta _{b}=\max \left\{ \left| \Delta _{\min }\right| ,\left| \Delta _{\max }\right| \right\} \end{aligned}$$

with

6 $$\begin{aligned} \Delta _{\min }=\frac{C_{m}}{x_{1M}+C_{m}T_{p}}-p_{0} \end{aligned}$$

and

7 $$\begin{aligned} \Delta _{\max }=\frac{C_{M}}{x_{1m}+C_{M}T_{p}}-p_{0} \end{aligned}$$

Then, the system (1) is changed to be

8 $$\begin{aligned} \left\{ \begin{array}{l} {\dot{x}}_{1}=N\left( p_{0}+\Delta \right) x_{2}-C \\ {\dot{x}}_{2}={-}\frac{x_{2}^{2}}{2}\left( p_{0}+\Delta \right) u_{r}+p_{0}+\Delta \\ y^{*}=x_{1} \end{array} \right. \end{aligned}$$

### *Remark 2*

It is easy to check that the range of $\Delta$ is much smaller than the bound of $\frac{C}{x_{1}+CT_{p}}$. Besides, all the link bandwidth *C* are unknown, such that the contradiction in^36,37^ does not exist in this paper.

### Assumption 2

Suppose that the continuity and boundedness of ideal trajectory vector ${\bar{y}}_{d}(t)=[y_{d}(t)$, ${\dot{y}}_{d}(t)$, $\ldots$, $y_{d}^{(n)}(t)]^{T}$ are guaranteed.

Different from the existing dynamics of uncertain TCP/AQM networks, the virtual control coefficients in this paper are divided into the known item $p_{0}$ and unknown bounded item $\Delta$. There exist ${\varepsilon } _{r1}N\Delta x_{2}$, ${\varepsilon }_{r2}\frac{\partial {\rho }_{r1}}{x_{1}} N\Delta x_{2}$, ${\varepsilon }_{r2}\Delta v_{r}$ and ${\varepsilon } _{r2}\Delta$ inevitably in the following controller design, which will be explained later. It is a severe challenge to deal with the above items.

## Controller design and main results

The following coordinate transformation is employed to design a controller via backstepping.

9 $$\begin{aligned} \varepsilon _{r1}=x_{1}-y_{d}^{*},\varepsilon _{r2}=x_{2}-\rho _{r1} \end{aligned}$$

***Step 1***: Select the Lyapunov function candidate as

10 $$\begin{aligned} V_{r1}=\frac{1}{2}{\varepsilon }_{r1}^{2}+\frac{1}{2q_{r}}{\tilde{C}}^{2} \end{aligned}$$

where $q_{r}>0$ is a constant and ${\tilde{C}}=C-C^{*}$ with $C^{*}$ being the approximation of *C*.

${\dot{V}}_{r1}$ is deduced as

11 $$\begin{aligned} {\dot{V}}_{r1}= & \,{\varepsilon }_{r1}{{\dot{\varepsilon }}}_{r1}-\frac{1}{q_{r}} {\tilde{C}}{\dot{C}}^{*} \nonumber \\= & \, {\varepsilon }_{r1}\left( N\left( p_{0}+\Delta \right) x_{2}-C-{\dot{y}} _{d}^{*}\right) -\frac{1}{q_{r}}{\tilde{C}}{\dot{C}}^{*} \nonumber \\= & \,{\varepsilon }_{r1}\left( Np_{0}\left( {\varepsilon }_{r2}+{\rho } _{r1}\right) -C-{\dot{y}}_{d}^{*}\right) +{\varepsilon }_{r1}N\Delta x_{2}- \frac{1}{q_{r}}{\tilde{C}}{\dot{C}}^{*} \nonumber \\= & \, Np_{0}{\varepsilon }_{r1}{\varepsilon }_{r2}+{\varepsilon }_{r1}\left( Np_{0}{\rho }_{r1}-C-{\dot{y}}_{d}^{*}\right) +{\varepsilon }_{r1}N\Delta x_{2}-\frac{1}{q_{r}}{\tilde{C}}{\dot{C}}^{*} \end{aligned}$$

Design the first virtual control signal as

12 $$\begin{aligned} {\rho }_{r1}=\frac{1}{Np_{0}}\left( -k_{r1}{\varepsilon }_{r1}+C^{*}+ {\dot{y}}_{d}^{*}\right) \end{aligned}$$

where $k_{r1}$ is a positive constant.

Invoking (12) leads to

13 $$\begin{aligned} {\dot{V}}_{r1}=-k_{r1}{\varepsilon }_{r1}^{2}+Np_{0}{\varepsilon }_{r1}{ \varepsilon }_{r2}+{\varepsilon }_{r1}N\Delta x_{2}+\frac{1}{q_{r}}{\tilde{C}} \left( \tau _{r1}-{\dot{C}}^{*}\right) \end{aligned}$$

where

14 $$\begin{aligned} \tau _{r1}=-q_{r}{\varepsilon }_{r1} \end{aligned}$$

***Step 2***: The 2th Lyapunov function candidate is given as

15 $$\begin{aligned} V_{r2}=V_{r1}+\frac{1}{2}{\varepsilon }_{r2}^{2} \end{aligned}$$

By some direct calculations, ${\dot{V}}_{r2}$ is written as

16 $$\begin{aligned} {\dot{V}}_{r2}= & {} {\dot{V}}_{r1}+{\varepsilon }_{r2}{{\dot{\varepsilon }}}_{r2} \nonumber \\= & {} -k_{r1}{\varepsilon }_{r1}^{2}+Np_{0}{\varepsilon }_{r1}{\varepsilon } _{r2}+{\varepsilon }_{r1}N\Delta x_{2}+\frac{1}{q_{r}}{\tilde{C}}\left( \tau _{r1}-{\dot{C}}^{*}\right) \nonumber \\{} & {} {-\varepsilon }_{r2}\frac{x_{2}^{2}}{2}\left( p_{0}+\Delta \right) u_{r}+{ \varepsilon }_{r2}\left( p_{0}+\Delta -{{\dot{\rho }}}_{r1}\right) \end{aligned}$$

Constructing

17 $$\begin{aligned} u_{r}=\frac{2}{x_{2}^{2}}v_{r} \end{aligned}$$

and substituting it in (16) yield

18 $$\begin{aligned} {\dot{V}}_{r2}= & {} -k_{r1}{\varepsilon }_{r1}^{2}+Np_{0}{\varepsilon }_{r1}{ \varepsilon }_{r2}+{\varepsilon }_{r1}N\Delta x_{2}+\frac{1}{q_{r}}{\tilde{C}} \left( \tau _{r1}-{\dot{C}}^{*}\right) \nonumber \\{} & {} {-\varepsilon }_{r2}\left( p_{0}+\Delta \right) v_{r}+{\varepsilon } _{r2}\left( p_{0}+\Delta -{{\dot{\rho }}}_{r1}\right) \nonumber \\= & {} -k_{r1}{\varepsilon }_{r1}^{2}+Np_{0}{\varepsilon }_{r1}{\varepsilon } _{r2}+{\varepsilon }_{r1}N\Delta x_{2}+\frac{1}{q_{r}}{\tilde{C}}\left( \tau _{r1}-{\dot{C}}^{*}\right) \nonumber \\{} & {} +{\varepsilon }_{r2}\left( {-}p_{0}v_{r}+p_{0}\right) -{\varepsilon }_{r2}{ {\dot{\rho }}}_{r1}-{\varepsilon }_{r2}\Delta v_{r}+{\varepsilon }_{r2}\Delta \end{aligned}$$

In view of the definition of $\rho _{r1}$ in (12), we have

19 $$\begin{aligned} {{\dot{\rho }}}_{r1}= & {} \frac{\partial {\rho }_{r1}}{x_{1}}{\dot{x}}_{1}+\frac{ \partial {\rho }_{r1}}{y_{d}^{*}}{\dot{y}}_{d}+\frac{\partial {\rho }_{r1} }{{\dot{y}}_{d}^{*}}\ddot{y}_{d}+\frac{\partial {\rho }_{r1}}{C^{*}} {\dot{C}}^{*} \nonumber \\= & {} \frac{\partial {\rho }_{r1}}{x_{1}}\left( N\left( p_{0}+\Delta \right) x_{2}-C\right) +F+\frac{\partial {\rho }_{r1}}{C^{*}}{\dot{C}}^{*} \end{aligned}$$

with

$$\begin{aligned} F=\frac{\partial {\rho }_{r1}}{y_{d}^{*}}{\dot{y}}_{d}+\frac{\partial { \rho }_{r1}}{{\dot{y}}_{d}^{*}}\ddot{y}_{d} \end{aligned}$$

Replacing ${{\dot{\rho }}}_{r1}$ in (18) by (19) produces

20 $$\begin{aligned} {\dot{V}}_{r2}= & {} -k_{r1}{\varepsilon }_{r1}^{2}+Np_{0}{\varepsilon }_{r1}{ \varepsilon }_{r2}+{\varepsilon }_{r1}N\Delta x_{2}+\frac{1}{q_{r}}{\tilde{C}} \left( \tau _{r1}-{\dot{C}}^{*}\right) \nonumber \\{} & {} +{\varepsilon }_{r2}\left( {-}p_{0}v_{r}+p_{0}-\frac{\partial {\rho }_{r1} }{x_{1}}\left( Np_{0}x_{2}-C^{*}\right) -F-\frac{\partial {\rho }_{r1}}{ C^{*}}{\dot{C}}^{*}\right) \nonumber \\{} & {} -{\varepsilon }_{r2}\left( \frac{\partial {\rho }_{r1}}{x_{1}}N\Delta x_{2} {+}\Delta v_{r}-\Delta -{\tilde{C}}\frac{\partial {\rho }_{r1}}{x_{1}}\right) \nonumber \\= & {} -k_{r1}{\varepsilon }_{r1}^{2}+Np_{0}{\varepsilon }_{r1}{\varepsilon } _{r2}+{\varepsilon }_{r1}N\Delta x_{2}+{\varepsilon }_{r2}\left( {-} p_{0}v_{r}+p_{0}-\frac{\partial {\rho }_{r1}}{C^{*}}{\dot{C}}^{*}\right) \nonumber \\{} & {} +\frac{1}{q_{r}}{\tilde{C}}\left( \tau _{r1}+q_{r}{\varepsilon }_{r2}\frac{ \partial {\rho }_{r1}}{x_{1}}-{\dot{C}}^{*}\right) -{\varepsilon }_{r2} \frac{\partial {\rho }_{r1}}{x_{1}}\left( Np_{0}x_{2}-C^{*}\right) \nonumber \\{} & {} -{\varepsilon }_{r2}F-{\varepsilon }_{r2}\frac{\partial {\rho }_{r1}}{x_{1} }N\Delta x_{2}-{\varepsilon }_{r2}\Delta v_{r}+{\varepsilon }_{r2}\Delta \end{aligned}$$

Design the following function

21 $$\begin{aligned} h_{r}(Z)=\Delta _{b}\left( \left| {\varepsilon }_{r1}Nx_{2}\right| +\left| {\varepsilon }_{r2}\frac{\partial {\rho }_{r1}}{x_{1}} Nx_{2}\right| +\left| {\varepsilon }_{{r}2}\right| \right) +\Delta _{b}\left| {\varepsilon }_{r2}\right| v_{r\max } \end{aligned}$$

where $v_{r\max }$ is the practical maximum of $v_{r}$, and $Z=\left[ x_{1},x_{2},y_{d}^{*},{\dot{y}}_{d}^{*},C^{*}\right] ^{T}$.

Then, choose the control law as

22 $$\begin{aligned} v= & {} {-}\frac{1}{p_{0}}\left( -k_{r2}{\varepsilon }_{r2}-Np_{0}{\varepsilon } _{r1}+\frac{\partial {\rho }_{r1}}{x_{1}}\left( Np_{0}x_{2}-C^{*}\right) -p_{0}F\right) \nonumber \\{} & {} {-}\frac{1}{p_{0}}\left( \frac{\partial {\rho }_{r1}}{C^{*}}\tau _{r2}- \frac{{\varepsilon }_{r2}h_{r}(Z)}{\Psi _{r}^{2}+{\varepsilon }_{r2}^{2}} \right) \end{aligned}$$

and

23 $$\begin{aligned} {\dot{\Psi }}_{r}=\left\{ \begin{array}{l} -\frac{\Psi _{r}h_{r}(Z)}{\Psi _{r}^{2}+{\varepsilon }_{r2}^{2}}-k_{rf}\Psi _{r},\left| {\varepsilon }_{ri}\right| \ge \sigma _{ri}\text { or } C^{*}\ge {\hat{C}} \\ 0,\text { else} \end{array} \right. \end{aligned}$$

where $k_{rf}$, $c_{r1}$ and $c_{r2}$ are positive constants and $c_{r1}<c_{r2}/k_{rf}$, $\Psi _{r}\left( 0\right)$ is the initial value of $\Psi _{r}$, which is chosen in the interval of $\left[ c_{r1},c_{r2}/k_{rf} \right]$, $i=1,2$, and

24 $$\begin{aligned} \tau _{r2}=\tau _{r1}+q_{r}{\varepsilon }_{r2}\frac{\partial {\rho }_{r1}}{ x_{1}} \end{aligned}$$

### *Remark 3*

Although there is ${{\dot{\rho }}}_{r1}$ in the controller (22), the repeated differentiation will not occur due to the system order only being 2. Besides, the proposed control scheme does not utilize approximators based on fuzzy logics or neural networks, which means that the computational complexity does not increase exponentially as the number of the rules increases. Therefore, the explosion of computation does nor exist. At worst, there have been many methods to deal with the problem^38^.

Substituting (22) in (20) results in

25 $$\begin{aligned} {\dot{V}}_{r2}= & {} -\sum _{i=1}^{2}k_{ri}{\varepsilon }_{ri}^{2}+{\varepsilon } _{r1}N\Delta x_{2}+\frac{1}{q_{r}}{\tilde{C}}\left( \tau _{r2}-{\dot{C}}^{*}\right) +{\varepsilon }_{r2}\frac{\partial {\rho }_{r1}}{C^{*}}\left( \tau _{r2}-{\dot{C}}^{*}\right) \nonumber \\{} & {} -{\varepsilon }_{r2}\frac{\partial {\rho }_{r1}}{x_{1}}N\Delta x_{2}-{ \varepsilon }_{r2}\Delta v_{r}+{\varepsilon }_{r2}\Delta -\frac{{\varepsilon }_{r2}^{2}h_{r}({Z})}{\Psi _{r}^{2}+{\varepsilon }_{r2}^{2}} \end{aligned}$$

Construct the adaptive law as

26 $$\begin{aligned} {\dot{C}}^{*}=\tau _{r2} \end{aligned}$$

By invoking (26) and considering (5), ${\dot{V}}_{r2}$ is rewritten as

27 $$\begin{aligned} {\dot{V}}_{r2}= & {} -\sum _{i=1}^{2}k_{ri}{\varepsilon }_{ri}^{2}+{\varepsilon } _{r1}N\Delta x_{2}-{\varepsilon }_{r2}\frac{\partial {\rho }_{r1}}{x_{1}} N\Delta x_{2}-{\varepsilon }_{r2}\Delta v_{r}+{\varepsilon }_{r2}\Delta - \frac{{\varepsilon }_{r2}^{2}h_{r}({Z})}{\Psi _{r}^{2}+{\varepsilon } _{r2}^{2}} \nonumber \\\le & {} \Delta _{b}\left( \left| {\varepsilon }_{r1}Nx_{2}\right| +\left| {\varepsilon }_{r2}\frac{\partial {\rho }_{r1}}{x_{1}} Nx_{2}\right| +\left| {\varepsilon }_{r2}v_{r}\right| +\left| {\varepsilon }_{r2}\right| \right) -\sum _{i=1}^{2}k_{ri}{ \varepsilon }_{ri}^{2}-\frac{{\varepsilon }_{r2}^{2}h_{r}(Z)}{\Psi _{r}^{2}+{ \varepsilon }_{r2}^{2}} \end{aligned}$$

The main results of the paper are stated below.

### Theorem 1

*Considering the nonlinear system* (1) *with Assumption* 1, *the developed control strategy, including the control law* (17) *and adaptive law* (26), *guarantees the practical boundedness of all the signals in the resulting closed-loop systems*.

### *Proof*

Design the 3th Lyapunov synthesis candidate as

28 $$\begin{aligned} V_{r3}=V_{r2}+\frac{1}{2}\Psi _{r}^{2} \end{aligned}$$

Case 1: $$\left| \varepsilon _{ri}\right| \ge \sigma _{ri}$$ or $$C^{*}\ge {\hat{C}}$$, $$i=1$$, 2

Then, ${\dot{V}}_{r3}$ is computed as

29 $$\begin{aligned} {\dot{V}}_{r3}= & {} {\dot{V}}_{r2}+\Psi _{r}{\dot{\Psi }}_{r} \nonumber \\\le & {} \Delta _{b}\left( \left| {\varepsilon }_{r1}Nx_{2}\right| +\left| {\varepsilon }_{r2}\frac{\partial {\rho }_{r1}}{x_{1}} Nx_{2}\right| +\left| {\varepsilon }_{r2}v_{r}\right| +\left| {\varepsilon }_{r2}\right| \right) -\sum _{i=1}^{2}k_{ri}{ \varepsilon }_{ri}^{2}-\frac{{\varepsilon }_{r2}^{2}h_{r}(Z)}{\Psi _{r}^{2}+{ \varepsilon }_{r2}^{2}}+\Psi _{r}{\dot{\Psi }}_{r} \nonumber \\= & {} \Delta _{b}\left( \left| {\varepsilon }_{r1}Nx_{2}\right| +\left| {\varepsilon }_{r2}\frac{\partial {\rho }_{r1}}{x_{1}} Nx_{2}\right| +\left| {\varepsilon }_{r2}v_{r}\right| +\left| {\varepsilon }_{r2}\right| \right) -\sum _{i=1}^{2}k_{ri}{ \varepsilon }_{ri}^{2} \nonumber \\{} & {} -\frac{\left( {\varepsilon }_{r2}^{2}+\Psi _{r}^{2}\right) h_{r}(Z)}{\Psi _{r}^{2}+{\varepsilon }_{r2}^{2}}-k_{rf}\Psi _{r}^{2} \nonumber \\= & {} -\sum _{i=1}^{2}k_{ri}{\varepsilon }_{ri}^{2}-k_{rf}\Psi _{r}^{2}-\Delta _{b}\left( v_{r\max }-\left| v_{r}\right| \right) \left| { \varepsilon }_{r2}\right| \nonumber \\\le & {} -\sum _{i=1}^{2}k_{ri}{\varepsilon }_{ri}^{2}-k_{rf}\Psi _{r}^{2}\le 0 \end{aligned}$$

It is known from (29) that $V_{r3}$ is bounded, which means that $\varepsilon _{r1}$, $\varepsilon _{r2}$, $C^{*}$, and $\Psi _{r}$ are all bounded. From $\varepsilon _{r1}=x_{1}-y_{d}^{*}$ and the boundedness of $y_{d}^{*}$, it is obtained that $x_{1}$ is bounded. According to (12), the boundedness of $\rho _{r1}$ is verified, which implies that $x_{2}$ is bounded by considering $\varepsilon _{r2}=x_{2}-\rho _{r1}$. It follows that $u_{r}$ is bounded. Therefore, the conclusion as Theorem 1 is obtained.

Case 2: $$\left| \varepsilon _{ri}\right| <\sigma _{ri}$$ and $$C^{*}<{\hat{C}}$$

It can not be obtained that ${\dot{V}}_{r3}\le -\kappa V_{r3}+\gamma$, which means $\varepsilon _{i}$ and $C^{*}$ may not be convergent. However, Case 2 will be switched to Case 1 when $\left| \varepsilon _{ri}\right| \ge \sigma _{ri}$ or $C^{*}\ge {\hat{C}}$, such that the practical boundedness of all the signals is obtained. $\square$

### *Remark 4*

(1) The range of $k_{ri}$ is wide. The bigger the parameter $k_{ri}$, the faster the response and smaller the steady state error. However, it cannot be too big. (2) $k_{rf}$ should be small enough and chosen according to the value of $c_{r2}$, such that $c_{r2}/k_{rf}$ is not too big. (3) ${q}_{r}$ should be big enough, which can guarantee that the steady state error is small enough. (4) $c_{r1}$ and $c_{r2}$ should satisfy $c_{r1}<c_{r2}/k_{rf}$, and $c_{r2}$ can not be selected too big, which may cause a bigger steady state error.

## Simulation results

In this section, the proposed control scheme is simulated for an uncertain TCP/AQM network and compared with the controller in^37^, which are abbreviated to be *C*2 and *C*1, respectively.

The uncertain TCP/AQM network is described as (1), whose parameters are chosen as $N=100$, $T_{p}=0.1$ s, $x_{1m}=100$ packets, $x_{10}=100$ packets, $x_{1M}=102$ packets, $x_{2M}=6$ byte, $C_{m}=1950$ packets/s, $C_{0}=2000$ packets/s, $C_{M}=2050$ packets/s. The saturation of $u_{r}$ is selected to be 1.

The initial states are given as $[x_{1}$, $x_{2}$, $\Psi _{r}$, $C^{*}]^{T}=[100$, 5.2, 3, $2000]^{T}$. In order to further show the superiority of *C*2, the following dynamic desired trajectory is chosen

$$\begin{aligned} y_{d}^{*}=\left\{ \begin{array}{l} 0.2(t-3)^{2}+100,0\le t\le 3s \\ 100,3s<t\le 7s \\ 0.1(t-7)^{2}+100,7s<t\le 10s \end{array},\right. \end{aligned}$$

which is always selected to be constant in^33–37^.

The virtual control signal $\rho _{r1}$, real control law $u_{r}$ and ${\dot{\Psi }}_{r}$ are constructed as (12), (17) and (23), with $k_{r1}=500$, $k_{r2}=500$, $k_{rf}=10$, $c_{r1}=1$, $c_{r2}=100$. The adaptive law ${\dot{C}}^{*}$ is designed as (26) with $q_{r}=5$.

**Figure 1:**
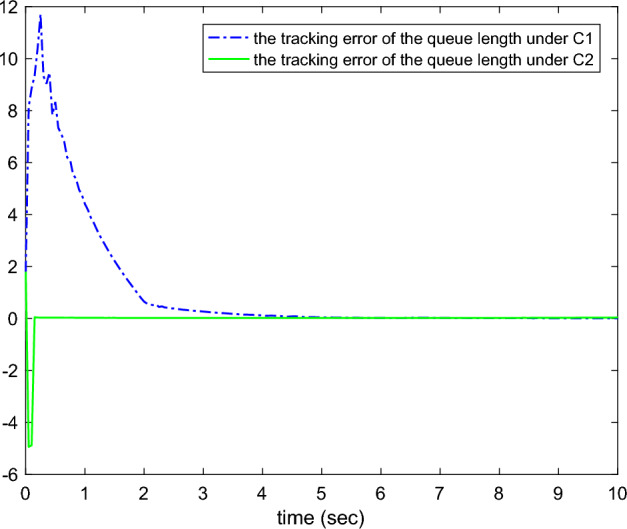
The tracking errors of the queue length.

**Figure 2:**
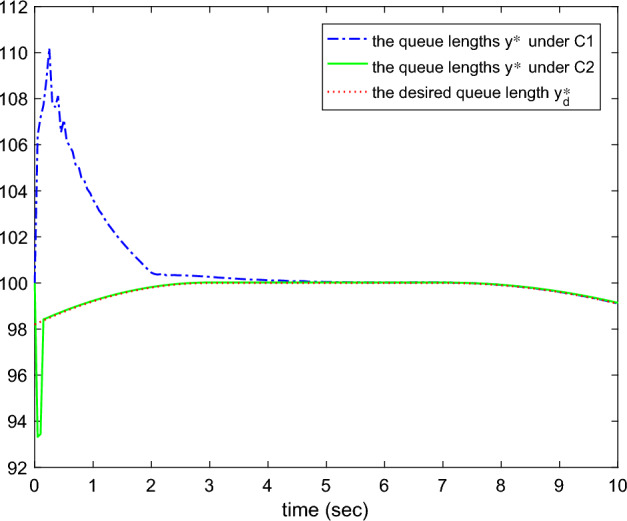
The actual and desired queue lengths.

**Figure 3:**
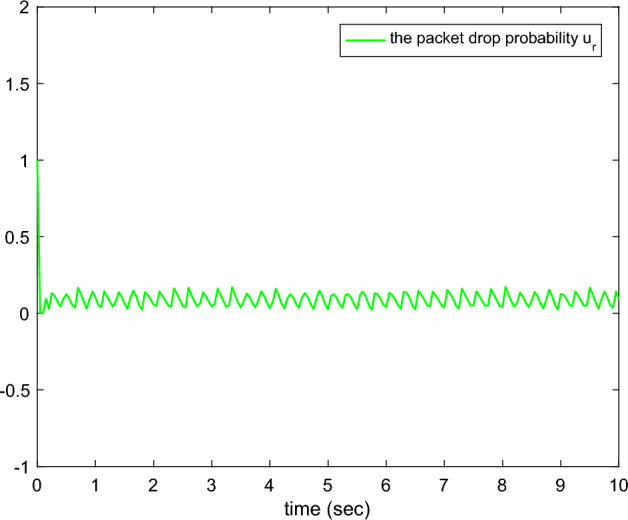
The packet drop probability.

**Figure 4:**
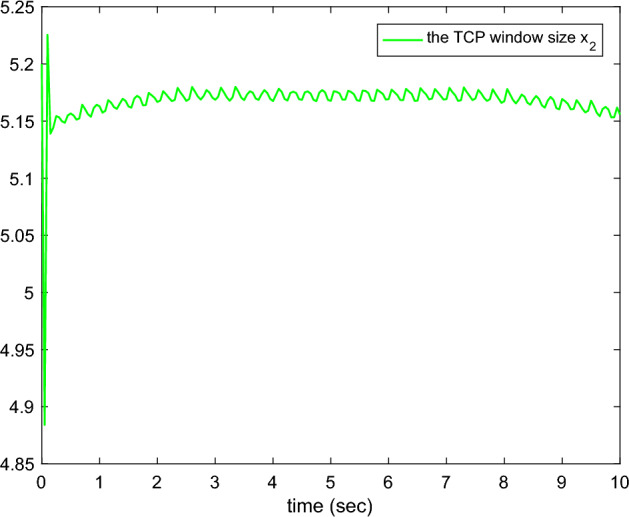
The TCP window size.

**Figure 5:**
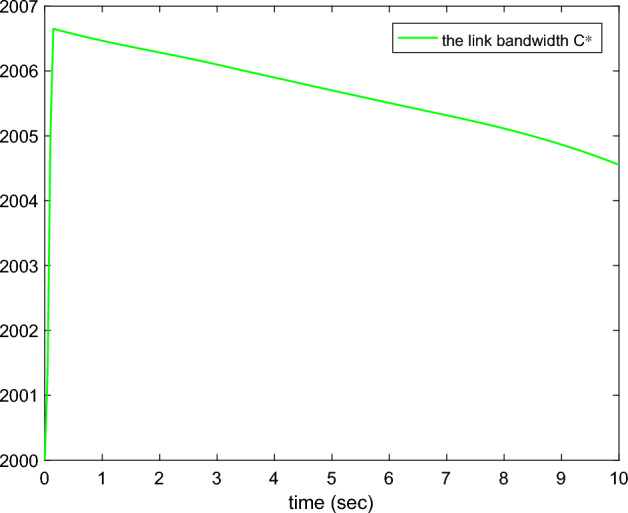
The adaptive link bandwidth.

**Figure 6:**
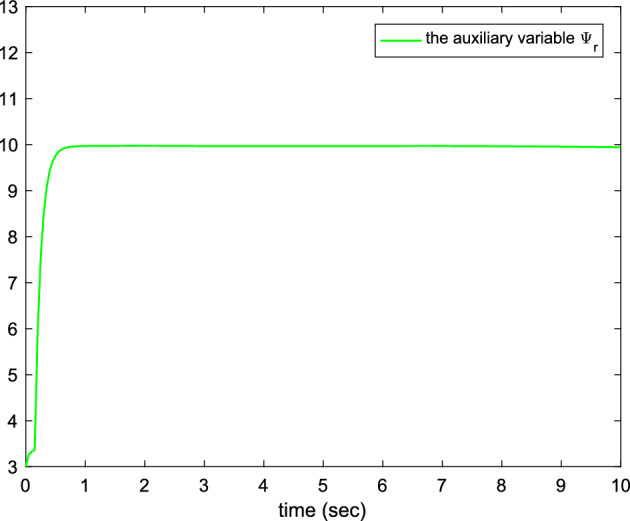
The auxiliary variable.

Figures 1, 2, 3, 4, 5 and reff6 show the simulation results. In Fig. 1, the tracking errors under *C*2 and *C*1 of the queue length are shown. It is easy to see that the error under *C*2 is smaller than the error under *C*1, whose absolute mean values are 0.0824, 1.0564 and root mean square values are 0.5063, 2.5891, respectively. The actual queue lengths $y^{*}$ are displayed in Fig. 2, which can track desired queue length $y_{d}^{*}$, after transient oscillation. However, the tracking performance under *C*2 is much better than *C*1 during the transient state. Figure 3 gives the packet drop probability $u_{r}$, which varies between 0 and 1 in the transient state and varies around 0.1 in the steady state. The TCP window size $x_{2}$ is drawn in Fig. 4, which oscillates before 0.2*s* and varies according to $y_{d}^{*}$. Figure 5 presents the adaptive link bandwidth $C^{*}$, it can be known that it tends to its real value 2000*b*/*s*. The auxiliary variable $\Psi _{r}$ is presented in Fig. 6, which is bounded and converges to 10.

## Conclusion

This paper has been devoted to the study of adaptive tracking control for nonlinear systems in a new form, whose virtual control coefficients consist of known and unknown items. The proposed controller not only utilized known information fully to pursue better control performance, but also handled unknown items by defining a novel auxiliary system. To demonstrate the feasibility of the developed control scheme, it was further applied to the congestion control of an uncertain TCP/AQM network system. In future, we plan to combine the proposed control scheme with fixed-time control^39,40^ and apply it to some other real systems, such as robots, quadrotors, and so on.

## Data Availability

All data generated or analysed during this study are included in this published article.
